# Artificial intelligence driven innovations in biochemistry: A review of emerging research frontiers

**DOI:** 10.17305/bb.2024.11537

**Published:** 2025-01-14

**Authors:** Mohammed Abdul Lateef Junaid

**Affiliations:** 1 Department of Basic Medical Sciences, College of Medicine, Majmaah University, Al Majmaah, Kingdom of Saudi Arabia

**Keywords:** Artificial intelligence, AI, biochemistry, machine learning, molecular modeling, drug discovery, personalized medicine, synthetic biology

## Abstract

Artificial intelligence (AI) has become a powerful tool in biochemistry, greatly enhancing research capabilities by enabling the analysis of complex datasets, predicting molecular interactions, and accelerating drug discovery. As AI continues to evolve, its applications in biochemistry are poised to expand, revolutionizing both theoretical and applied research. This review explores current and potential AI applications in biochemistry, with a focus on data analysis, molecular modeling, enzyme engineering, and metabolic pathway studies. Key AI techniques—such as machine learning algorithms, natural language processing, and AI-based molecular modeling—are discussed. The review also highlights emerging research areas benefiting from AI, including personalized medicine and synthetic biology. The methodology involves an extensive analysis of existing literature, particularly peer-reviewed studies on AI applications in biochemistry. AI-driven tools like AlphaFold, which have significantly advanced protein structure prediction, are evaluated alongside AI’s role in expediting drug discovery. The review also addresses challenges, such as data quality, model interpretability, and ethical considerations. Results indicate that AI has expanded the scope of biochemical research by facilitating large-scale data analysis, enhancing molecular simulations, and opening new avenues of inquiry. However, challenges remain, particularly in data handling and ethical concerns. In conclusion, AI is transforming biochemistry by driving innovation and expanding research possibilities. Future advancements in AI algorithms, interdisciplinary collaboration, and integration with automated techniques will be crucial to fully unlocking AI’s potential in advancing biochemical research.

## Introduction

Research combining artificial intelligence (AI) and biochemistry has rapidly progressed over the past decade, transforming the field. Initial AI applications were primarily in bioinformatics, utilizing machine learning algorithms to analyze large-scale genomic and proteomic data, setting the stage for more sophisticated uses [1]. These developments have enabled AI-driven molecular modeling and drug discovery, particularly in understanding protein structures and interactions [2]. A major breakthrough, AlphaFold by DeepMind, has achieved exceptional accuracy in protein folding predictions, addressing a longstanding challenge in structural biology [3]. Beyond structural predictions, AI is increasingly applied in enzyme engineering and metabolic pathway analysis, facilitating enzyme design with enhanced functionality and enabling researchers to predict outcomes of complex biochemical reactions [4]. Combining AI with high-throughput screening techniques has significantly reduced the time and costs associated with traditional drug discovery methods [5]. Such advancements highlight AI’s critical role in expanding the potential of biochemical research.

### AI

AI refers to the simulation of human intelligence by machines. It encompasses a broad range of techniques and algorithms that enable machines to perform tasks requiring human-like reasoning, learning, and decision-making. AI serves as the overarching field that includes various subfields, such as machine learning and deep learning [6].

### Machine learning

It is a subset of AI focused on developing algorithms that enable machines to learn from and make predictions or decisions based on data. Unlike traditional programming, where explicit instructions are provided for every task, machine learning models improve their performance over time as they process and analyze more data [7].

### Deep learning

It is a specialized branch of machine learning that uses artificial neural networks with multiple layers (hence the term “deep”) to model complex patterns in large datasets. DL has been especially impactful in fields, such as image recognition, natural language processing, and biochemistry, enabling advanced tasks like protein structure prediction [7].

### Generative adversarial networks (GANs)

GANs are a class of deep learning models consisting of two neural networks—a generator and a discriminator—that work in opposition. The generator creates new data samples (e.g., molecular structures), while the discriminator evaluates the quality of these samples. This adversarial process enables GANs to generate highly realistic and innovative outputs, such as designing novel molecules for drug discovery [7].

### Current state and rationale

Currently, AI-driven biochemistry represents a dynamic and rapidly evolving field where AI tools not only provide critical support but also revolutionize the methods employed in biochemical research. For instance, AI has proven instrumental in addressing challenges once deemed insurmountable, such as achieving accurate protein structure predictions with AlphaFold and streamlining the drug discovery process [8]. By processing vast datasets and generating predictive models, AI has significantly expanded our understanding of molecular interactions, enzyme functions, and metabolic pathways [9]. Additionally, it is reshaping experimental biochemistry by improving the precision and efficiency of experimental design and execution [10].

### Published meta-analyses data related to AI applications in biochemistry

To date, a limited number of meta-analyses have been conducted in the field of AI applications in biochemistry. While some studies have systematically reviewed specific subfields, such as AI-driven drug discovery [11] or protein structure prediction [12], comprehensive meta-analyses integrating diverse applications of AI across all major biochemical domains remain scarce. For example, prior meta-analyses have primarily focused on individual topics, such as the accuracy of AI algorithms in predicting protein structures or the efficiency of AI models in virtual drug screening. Although these studies are valuable, they fail to address the broader implications and interdisciplinary applications of AI in biochemistry. This review aims to fill this gap by providing an extensive narrative synthesis of recent advancements, tools, and applications of AI in biochemistry, including its role in enzyme engineering, metabolic pathway modeling, and synthetic biology. Furthermore, it identifies key challenges and outlines future directions to encourage further research in this transformative field. By synthesizing insights across multiple subfields, this review offers a holistic perspective that complements and expands upon the scope of existing meta-analyses. In recent years, AI has experienced rapid advancements, significantly broadening its impact across diverse areas of biochemistry. Below are some noteworthy developments.

### AI in protein structure prediction

Proteins are at the core of most biological processes. Because the function of a protein depends on its structure, understanding protein structures has been a grand challenge in biology for decades. Although several experimental techniques for structure determination have been developed and their accuracy improved, these methods remain both difficult and time-consuming. As a result, decades of theoretical work have focused on predicting protein structures from amino acid sequences. Recently, AI-based tools like AlphaFold have achieved groundbreaking success in this area. Jumper et al. [2] (2021) demonstrated that AlphaFold can predict protein folding with near-experimental accuracy, addressing one of the most challenging problems in structural biology.

### Drug discovery and virtual screening

Drug discovery is the process of identifying and developing new medications to treat diseases. It involves various stages, including target identification, where specific molecules related to a disease are selected; lead discovery, where potential drug compounds are identified; and optimization, where these compounds are refined for better efficacy and safety. This process combines biology, chemistry, and technology, and typically requires years of research and testing before a drug can be approved for use in patients. Drug discovery aims to find treatments that improve health outcomes and offer new solutions to unmet medical needs. AI has revolutionized drug discovery by expediting the identification of potential drug candidates. For instance, Stokes et al. (2020) employed deep learning to discover Halicin, a novel antibiotic effective against drug-resistant bacteria. AI-driven platforms like Atomwise and Schrödinger have further accelerated the virtual screening of millions of compounds [8].

### Enzyme engineering

Enzyme engineering is the process of designing and modifying enzymes to improve their properties or create new functions. This field combines techniques from molecular biology, protein chemistry, and biochemistry to alter the structure of enzymes, enhancing their stability, specificity, or catalytic efficiency for various industrial, medical, and environmental applications. Engineered enzymes have a wide range of uses, including drug development, biofuel production, and the food industry. By optimizing enzymes for specific tasks, enzyme engineering helps develop more efficient and sustainable solutions for many challenges. AI models are increasingly utilized in enzyme design, optimizing catalytic properties and enhancing stability. For example, Ryu et al. [13] (2019) demonstrated the use of neural networks to predict enzyme commission (EC) numbers, paving the way for more efficient enzyme engineering in industrial biotechnology.

### Metabolic pathway analysis and synthetic biology

Machine learning techniques are advancing our understanding of metabolic pathways. Cheng et al. [14] (2023) highlighted how AI can predict missing enzymes and metabolites, enabling the design of synthetic biological systems for applications, such as biofuels and biopharmaceuticals.

### Integration of multi-omics data

Recent studies have focused on integrating multi-omics data (e.g., genomics, proteomics, and metabolomics) using AI algorithms to uncover complex biological interactions. For instance, Vasaikar et al. [15] (2018) demonstrated how AI-driven analysis of multi-omics datasets could unravel the biochemical underpinnings of diseases, with a particular emphasis on advancing cancer research.

### Personalized medicine

AI is revolutionizing personalized medicine by analyzing patient-specific genetic and biochemical data to tailor treatments more effectively. Mamoshina et al. [16] (2018) demonstrated how AI aids in the discovery of biomarkers and tissue-specific drug targets, driving advancements in precision healthcare.

### Emerging AI techniques

GAN and reinforcement learning are now being used to design novel molecules and optimize metabolic pathways. For instance, Zeng et al. [17] (2022) demonstrated how GANs can accelerate the discovery of chemical compounds with desired properties, significantly shortening experimental timelines. By synthesizing these advancements, this review offers readers a comprehensive overview of the latest progress in the field. The rapid evolution of AI continues to reshape biochemistry, underscoring the importance for researchers to remain informed about these cutting-edge developments. However, significant challenges remain in fully harnessing AI’s potential in healthcare research. While many AI models are powerful, they often struggle with issues such as interpretability and the complexity of biological systems, which can lead to uncertainty in AI-generated predictions. Additionally, challenges persist with data quality, algorithmic biases, and the integration of AI with traditional biochemical approaches [18].

## Materials and methods

This review takes a comprehensive and structured approach to examine the role of AI in advancing biochemical research. The methodology is divided into three key stages: literature selection, data extraction, and the analysis of AI applications across diverse subfields of biochemistry. By following this process, the review not only showcases the latest advancements but also identifies challenges and explores potential future directions in the field.

### Literature selection

The first step involved a systematic search of peer-reviewed journals, conference papers, and authoritative reviews. Databases, such as PubMed, Google Scholar, and Scopus were utilized to retrieve relevant publications spanning the years 2000–2024. The primary search terms included “artificial intelligence in biochemistry,” “AI in drug discovery,” “machine learning in biochemistry,” “protein structure prediction with AI,” and “AI in metabolic pathways.” Boolean operators (AND, OR) were employed to refine search results and exclude irrelevant studies. Only studies published in English were considered for inclusion in this review.

### Inclusion and exclusion criteria

To ensure a focus on high-impact research, inclusion criteria were established based on relevance to AI and its applications in biochemistry. Papers were included if they directly explored the use of AI techniques—such as machine learning, neural networks, or natural language processing—in fields like drug discovery, molecular modeling, enzyme engineering, and metabolic pathway analysis. Papers that only discussed AI generically or lacked specific biochemical applications were excluded. Initially, 120 studies were identified. Following a screening of titles and abstracts for relevance, 80 papers were shortlisted for further review. Of these, 40 papers were ultimately selected for in-depth analysis, with a primary focus on key areas, such as drug discovery, molecular modeling, enzyme engineering, and metabolic pathway prediction.

**Figure 1.**
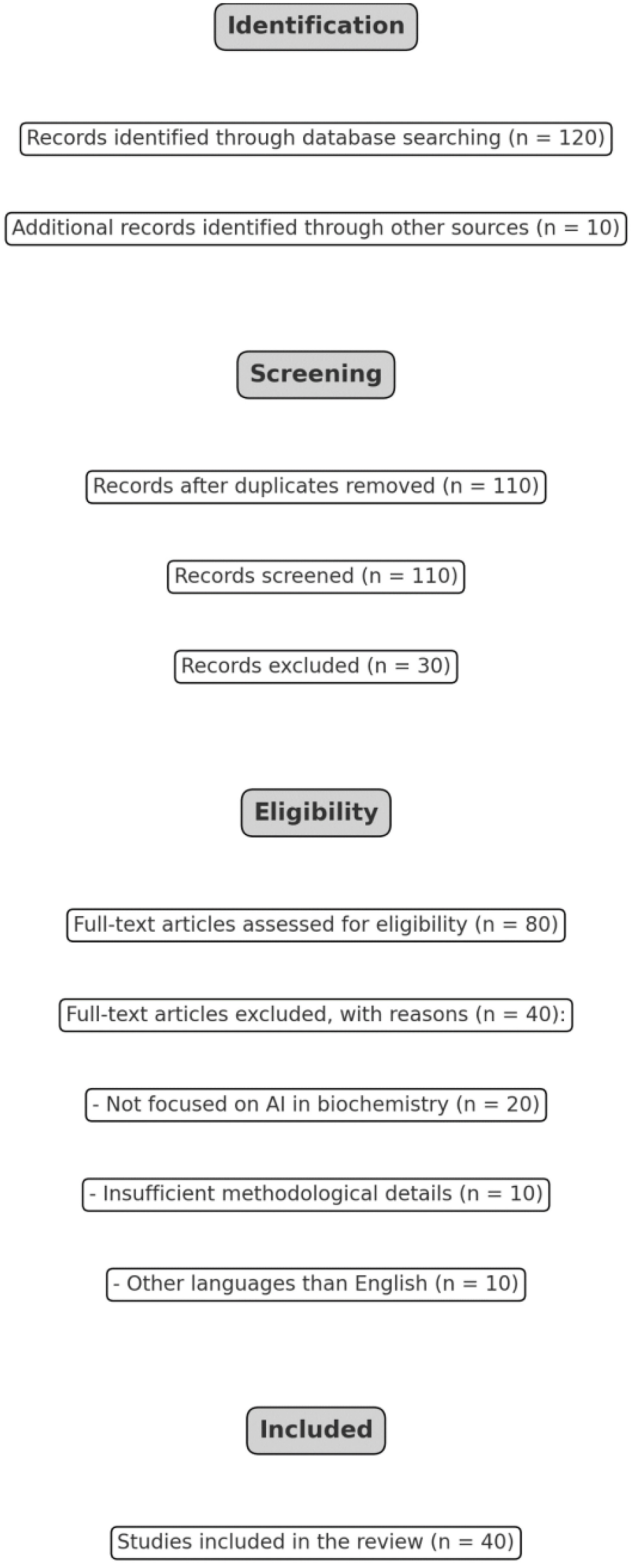
The PRISMA flowchart illustrates the systematic approach used to identify, screen, and include relevant studies for this review.

The PRISMA flowchart illustrating the systematic approach used to identify, screen, and include relevant studies for this review is shown in Figure 1.

### Data extraction and categorization

Data were extracted from selected studies focusing on AI applications in key areas, such as data analysis, molecular modeling, drug discovery, enzyme engineering, and synthetic biology. For each study, the following information was collected: i) the specific AI techniques used (e.g., deep learning, reinforcement learning, neural networks), ii) the biochemical problem addressed (e.g., protein structure prediction, drug target identification), iii) the results and impact of AI on biochemical research, and iv) the limitations and challenges faced in applying AI. The data were categorized by application area, enabling a structured analysis of AI’s impact across various subfields of biochemistry. The analysis aimed to identify trends in AI utilization, evaluate its effectiveness in solving biochemical problems, and highlight areas where AI has shown the greatest potential. Groundbreaking AI tools, such as AlphaFold for protein structure prediction [2] and AI-driven drug discovery platforms like Atomwise [8], were given special attention. AI applications in enzyme engineering were also reviewed, particularly their use in predicting enzyme-substrate interactions and optimizing enzyme design for industrial purposes [4]. In addition to the technical analysis, the study addressed current limitations of AI applications, including issues, such as data availability, model interpretability, and biases in AI algorithms. Ethical considerations, such as the risks of over-reliance on AI models and their implications for experimental reproducibility, were also explored [18]. This methodology ensures a balanced overview of the current state of AI in biochemistry while identifying gaps in knowledge and opportunities for future research.

## Results

### AI in protein structure prediction

The accurate prediction of protein structures has long been a cornerstone challenge in biochemistry, with profound implications for drug design and molecular biology. AlphaFold, developed by Jumper et al. (2021), represents a paradigm shift in structural biology, solving the decades-old problem of protein folding prediction. By accurately predicting the structures of over 98% of human proteins with remarkable precision, AlphaFold has unlocked new possibilities for modeling protein-ligand interactions—an essential step in accelerating drug discovery and therapeutic development [2]. Building on this success, Lin et al. [19] (2020) leveraged generative AI models to design novel peptides and proteins, expanding opportunities for therapeutic and industrial applications. This innovative approach enables the creation of targeted biomolecules with optimized properties for specific functions. Similarly, Pearce and Zhang (2021) advanced the field by applying deep learning techniques to predict protein–ligand binding sites, a critical factor in enhancing drug efficacy. Their work effectively bridges the gap between protein structure prediction and practical drug design, enabling the development of drugs that precisely target specific proteins [20]. Collectively, these studies underscore how AI-driven tools have revolutionized protein structure prediction, shifting the field from labor-intensive trial-and-error methods to precise, data-driven modeling. By deepening our understanding of molecular functions and interactions, AI not only improves protein structure prediction but also facilitates the design of targeted drugs and biomolecules. A notable trend across these advancements is the increasing focus on protein–ligand interactions, directly linking structural biology with practical applications in drug discovery.

### Significance and practical applications

AlphaFold’s ability to predict protein structures with high accuracy enables laboratories to model previously unsolved proteins, design targeted inhibitors, and investigate protein–protein interactions. In drug design, these insights play a pivotal role in advancing therapeutics aimed at specific proteins. By bridging critical gaps in understanding protein functions and interactions, AlphaFold has become instrumental in driving progress in drug discovery and enzyme engineering.

### Weaknesses and challenges

While AlphaFold excels in achieving structural accuracy, its utility in predicting protein dynamics and flexibility—key factors for drug binding and enzymatic activity—remains limited. Furthermore, its dependence on homologous protein databases diminishes its effectiveness when no similar structures are available. The reliance on high-quality input data, coupled with the “black box” nature of its neural network model, also restricts interpretability. Moreover, AlphaFold faces challenges in accurately predicting regions of proteins that lack homologous structures in its training datasets, raising concerns about its generalizability.

### Innovative opinion and future directions

Expanding AlphaFold’s capabilities to predict molecular dynamics and integrating its predictions with experimental structural biology techniques, such as cryo-EM, could help address current limitations. Future versions of AlphaFold might incorporate experimental feedback loops, allowing for iterative refinement of predictions. This approach could significantly improve the model’s robustness, particularly in scenarios where training data are limited or incomplete.

### AI in drug discovery

AI has revolutionized drug discovery by enabling rapid screening and identification of potential therapeutics. Recent advancements in AI have significantly reshaped this field, introducing innovative methods to identify novel compounds and predict complex biological interactions. A landmark example is the work of Stokes et al. (2020), who demonstrated the potential of deep learning by discovering Halicin—a groundbreaking antibiotic effective against drug-resistant bacteria. Their study, which involved screening millions of compounds in silico, showcased the power of AI to accelerate the identification of promising drug candidates [8]. Building on these advances, Zhou et al. (2024) applied reinforcement learning and neural networks to prioritize high-potential drug candidates from vast compound libraries, streamlining the preclinical development process. This methodology exemplifies how AI tools are optimizing the early stages of drug development, significantly reducing the time from discovery to clinical trials [21]. AI’s contributions extend well beyond drug discovery, finding applications in the design of complex biological systems. For instance, Qi et al. [22] highlighted AI’s transformative role in metabolic pathway design. By predicting enzymatic reactions, AI plays a vital role in synthetic biology applications, such as engineering microbial strains for biofuel production. This capability not only accelerates the development of bio-based solutions but also opens the door to innovations in personalized medicine.Furthermore, Jiménez-Luna et al. (2021) explored AI’s ability to predict drug-target interactions, enhancing the precision of virtual screening processes. Predicting the binding affinity of molecules to specific targets is crucial for identifying effective treatments and optimizing drug efficacy [23]. The versatility of AI in drug discovery is also evident in phenotypic drug discovery, as highlighted by Moffat et al. (2021). Their research underscores how AI, by analyzing complex biological systems, can identify drugs that affect multiple cellular processes. This capability is especially critical for addressing diseases with intricate mechanisms. The shift toward understanding drug effects through phenotypic responses represents a significant step beyond traditional target-based approaches [24].

Finally, Li and colleagues (2021) presented an insightful review on AI-driven structure-based drug design, emphasizing how AI models can predict structure-activity relationships. By enhancing the accuracy of molecular docking and identifying molecules with strong binding affinities to specific proteins, AI is revolutionizing drug development, making the process faster and more efficient [25]. Collectively, these studies highlight the wide-ranging applications of AI in drug discovery—from screening vast compound libraries to designing intricate metabolic pathways. The integration of AI not only accelerates the identification of novel drugs but also improves the precision and efficiency of every step in the drug development pipeline.

### Significance and practical applications

Tools like Halicin’s discovery highlight how AI can dramatically shorten the time needed to identify novel antibiotics or drug candidates. In laboratory settings, AI streamlines high-throughput screening, prioritizes molecules for synthesis, and predicts drug efficacy. In clinical settings, AI supports personalized treatment by forecasting patient-specific responses to medications. This approach reshapes the timeline and cost structure of antibiotic discovery, offering a scalable model to combat antimicrobial resistance.

### Weaknesses and challenges

Despite these advancements, clinical translation remains a significant hurdle. AI models often rely on chemical libraries that lack diversity, which limits the exploration of novel chemical spaces. Furthermore, ensuring the safety and efficacy of AI-predicted molecules in clinical trials continues to be both time-consuming and costly. Although these models have shown success, they heavily depend on training datasets curated from existing chemical libraries—datasets that may not include entirely novel chemical scaffolds. Additionally, the ethical implications of relying on AI-generated predictions without exhaustive validation present another layer of concern.

### Innovative opinion and future directions

Addressing these gaps requires improving the diversity of training datasets, integrating AI predictions with real-world experimental validation, and fostering stronger collaboration between computational researchers and clinical practitioners. By developing hybrid models that merge AI-driven predictions with rigorous experimental validation, researchers can enhance reproducibility, improve reliability, and address potential ethical concerns more effectively.

### AI in enzyme engineering

AI has become a cornerstone in advancing enzyme engineering, metabolic engineering, and drug development. For example, Ryu et al. (2019) demonstrated the potential of neural networks to predict EC numbers, enabling researchers to identify enzymes tailored for specific industrial applications. This method significantly reduces reliance on time-consuming trial-and-error approaches in enzyme engineering, paving the way for more efficient biotechnological processes [13]. Building on this foundation, Xie and Warshel [10] (2023) further optimized enzyme catalysis and stability using AI, accelerating progress by minimizing the need for extensive experimental trials. In the field of metabolic engineering, Cheng et al. [14] (2023) employed machine learning to predict the impact of genetic modifications on enzymatic activity. This innovative approach facilitates the precise design of metabolic networks, unlocking new opportunities to enhance industrial bioprocesses and advance synthetic biology. AI’s influence is equally profound in protein design. Fadahunsi et al. [26] explored AI’s ability to predict protein–ligand interactions, highlighting how AI streamlines drug discovery by identifying effective binding structures. Their findings underscore AI’s broader impact on both drug development and metabolic network optimization. Similarly, Quazi [27] demonstrated how machine learning models can predict the effects of genetic variations on metabolic pathways and biochemical behaviors. These predictive capabilities not only deepen our understanding of genotype–phenotype relationships but also hold immense promise for advancing personalized medicine and precision treatments. AI’s contributions to biological modeling and simulation have revolutionized the study of complex biological systems. By enhancing models of human cells and tissues, AI generates clearer hypotheses about metabolic pathways, driving progress in both research and clinical applications. For instance, Ebenhöh and Heinrich [28] investigated the use of AI-driven evolutionary algorithms to optimize metabolic pathways. Their work highlights the transformative potential of AI in industries, such as biofuel and biopharmaceutical production, where efficiency is critical.

In early drug development, AI’s transformative impact is highlighted by Serrano’s research [29], which focuses on biological target modeling and drug efficacy prediction. By leveraging AI-driven virtual screening and compound optimization, this work demonstrates how AI can significantly reduce reliance on traditional, resource-intensive *in vitro* and *in vivo* testing, thereby accelerating the drug discovery process. Similarly, Planes and Beasley [30] showcased how AI-driven optimization of metabolic pathways, using evolutionary algorithms, maximizes productivity in industrial biochemical processes such as biofuel production, further underscoring AI’s industrial relevance. Additionally, Paul et al. [31] reviewed AI’s role in pharmaceutical R&D, highlighting its ability to streamline drug discovery, lower costs, and expedite the development of new drugs by enabling early identification of therapeutic targets. Collectively, these studies illustrate the far-reaching impact of AI across various biochemical fields. From enzyme engineering to drug design, AI enhances our capacity to predict, optimize, and accelerate critical research processes, ultimately reducing both the time and costs traditionally required in these areas.

### Significance and practical applications

Laboratories leverage AI-predicted enzymes to develop more efficient biocatalysts, cutting costs and boosting yields in processes, such as drug synthesis and biofuel production. For instance, AI-designed enzymes optimize metabolic pathways in microorganisms used for industrial fermentation. By tailoring enzyme design for specific applications in biofuels, pharmaceuticals, and the food industry, AI is revolutionizing industrial biotechnology. Its predictive power enhances enzyme functionality and stability, driving innovation across multiple sectors.

### Weaknesses and challenges

Transitioning AI-designed enzymes to practical applications is a challenging process. Predicted enzymes often underperform in experimental systems because of unaccounted environmental variables that influence their functionality. Moreover, limitations in understanding enzyme-substrate specificity hinder the accuracy of AI tools, especially for enzymes involved in rare or poorly characterized reactions, where insufficient training data further reduces predictive precision. Compounding these challenges is the issue of reproducibility, as AI-designed enzymes may not consistently perform across different experimental setups.

### Innovative opinion and future directions

Combining AI predictions with iterative directed evolution experiments can significantly refine enzyme designs and improve their practical applications. By integrating AI-driven insights with directed evolution processes, researchers can enhance both the efficiency and reliability of enzyme engineering pipelines, leading to more robust and targeted results.

### AI in multi-omics and systems biology

AI tools are revolutionizing the understanding of complex biological systems by integrating diverse omics data. Their influence across biomedical research continues to grow, particularly in cancer research, metabolic engineering, drug repositioning, and systems biology. For example, Vasaikar et al. used AI to integrate and analyze multi-omics datasets, uncovering critical pathways linked to cancer progression. By combining genomics, proteomics, and metabolomics, this AI-driven approach is advancing precision oncology by revealing key insights into the molecular basis of cancer [15]. In another application, Cuperlovic–Culf employed machine learning to model metabolic pathways, enabling precise predictions of enzymatic reactions and metabolic flux. This computational strategy deepens our understanding of metabolic processes, paving the way for targeted interventions in metabolic disorders and innovations in biotechnology [32]. Broadening AI’s impact in molecular biology, Huang et al. (2023) developed a platform to explore microRNA-disease associations. This tool integrates and analyzes large-scale biological datasets, offering new insights into the role of miRNAs in disease progression and unlocking potential therapeutic opportunities [33]. During the COVID-19 pandemic, Richardson et al. (2020) demonstrated the power of AI in drug repositioning by predicting the effectiveness of existing drugs, such as Baricitinib, against COVID-19. By analyzing drug-target interactions, their work highlighted how AI can accelerate drug discovery and repurposing during public health emergencies [34].

In the context of gene-disease associations, Stoeger and colleagues (2020) utilized AI to analyze extensive datasets, uncovering patterns that clarified why certain genes have been overlooked in research. Their work addresses critical gaps in gene-disease research by shedding light on underexplored genetic factors [35]. Similarly, Ferrero et al. (2020) demonstrated AI’s utility in in silico prediction of therapeutic targets. By employing machine learning and knowledge graphs, they streamlined the identification of drug targets, significantly enhancing the efficiency of drug discovery [36]. AI’s potential in chemical synthesis was highlighted by Segler et al. (2020), who used deep neural networks to generate novel chemical synthesis pathways. This application optimizes the production of complex biochemical compounds, crucial for drug development and innovative therapies [37]. Likewise, Piñero et al. (2020) reviewed the DisGeNET platform, which integrates data on human disease-associated genes and variants. Their study demonstrated how AI facilitates the management and analysis of large genomic datasets, uncovering gene-disease relationships and advancing biomedicine and drug discovery [38]. Finally, Kim et al. (2020) showcased AI’s contributions to systems metabolic engineering, where AI-driven microbial strain design optimized biochemical production. Their findings underscore AI’s role in enhancing industrial biochemistry, with significant implications for pharmaceutical and bio-based chemical production [39]. Collectively, these studies highlight AI’s transformative impact across multiple domains of biochemical and medical research. From advancing precision oncology and metabolic engineering to optimizing drug repositioning and therapeutic target discovery, AI is driving efficiency, uncovering hidden patterns, and accelerating innovation in biomedicine.

### Significance and practical applications

In laboratories, AI facilitates the identification of biomarkers from multi-omics data, informs pathway analysis, and drives advancements in precision medicine. Clinically, AI supports personalized treatment planning by linking genomic variations to disease phenotypes. This approach reveals complex biological interactions that traditional analytical tools cannot uncover, paving the way for the identification of actionable biomarkers critical to personalized medicine.

### Weaknesses and challenges

A major challenge lies in the interpretability of AI-derived insights from complex datasets. Ensuring data quality and consistency across multi-omics platforms further compounds this difficulty. The analysis relies heavily on high-quality, large-scale omics data, which are not always accessible. Moreover, deciphering the biological significance of AI-derived patterns presents a significant obstacle, given the intricate nature of the models employed.

### Innovative opinion and future directions

Addressing these challenges requires standardizing data collection and processing methods, as well as developing interpretable AI models that deliver actionable insights. Integrating AI with pathway-based modeling can further improve biological interpretability, resulting in more practical and meaningful applications.

### Generative AI in molecule design

Recent advancements in AI have revolutionized the design of molecular structures and chemical compounds, accelerating the development of new drugs and biomolecules. Zeng et al. (2022) demonstrated the use of GANs to create molecular structures tailored to specific applications. This AI-driven approach minimizes reliance on traditional trial-and-error methods, significantly speeding up the discovery process for novel drugs and biomolecules [16]. Building on this, Visan and Nuget [40] highlight the transformative role of deep learning and AI in chemical compound discovery. Their research shows how AI-driven models, including GANs and reinforcement learning, can explore vast chemical spaces far more efficiently than conventional methods. These techniques enable the rapid identification of promising compounds, streamlining drug discovery, and chemical synthesis. Together, these studies illustrate the immense potential of AI in molecular design. By leveraging advanced models like GANs and reinforcement learning, AI is reducing the time and cost associated with discovering new chemical compounds, paving the way for a new era of rapid and efficient drug and biomolecule development.

### Significance and practical application

GANs can explore chemical spaces more effectively than traditional methods, enabling the generation of innovative molecular designs with potential applications in synthetic biology and pharmaceuticals.

### Weaknesses and challenges

GANs are prone to generating chemically unstable or biologically irrelevant molecules if the training dataset lacks sufficient diversity. Additionally, the interpretability of the generated designs continues to pose a significant challenge.

### Innovative opinion and future directions

Coupling GANs with in silico validation tools and experimental feedback mechanisms can significantly enhance the quality and practicality of generated molecules.

A brief summary of key studies leveraging AI-based approaches in computational biology, highlighting their application areas and impacts is shown in Table 1.

**Table 1:** An overview of AI-based approaches in computational biology and their applications

**Authors**	**Country**	**AI-based approach**	**Application area**	**Effect/Impact**
Jumper et al.	United Kingdom	Deep learning (AlphaFold)	Protein structure prediction	Achieved high accuracy in protein folding predictions, enabling drug design and understanding of molecular functions.
Stokes et al.	USA	Generative models (deep learning)	Drug discovery	Discovered new antibiotics (e.g., Halicin) through rapid screening of millions of compounds.
Ryu et al.	South Korea	Neural networks	Enzyme engineering	Predicted enzyme commission numbers with high accuracy, improving enzyme design for industrial applications.
Cheng et al.	China	Machine learning	Metabolic pathway modeling	Identified missing enzymes in metabolic networks, enhancing synthetic pathway reconstruction.
Zeng et al.	USA	Generative adversarial networks (GANs)	Drug design	Accelerated compound discovery by generating molecular structures with desired properties.

The above table summarizes key studies leveraging AI-based approaches in computational biology, highlighting their application areas and impacts. It includes examples of deep learning, generative models, neural networks, and machine learning techniques used for tasks such as protein structure prediction, drug discovery, enzyme engineering, metabolic pathway modeling, and drug design. The table illustrates the transformative potential of AI in accelerating research and innovation in biological sciences.

## Discussion

AI applications in biochemistry—spanning drug discovery, protein structure prediction, enzyme engineering, and metabolic optimization—have significantly reduced time and resource requirements. With its predictive accuracy and efficiency, AI is driving transformative advancements in biochemical research and industrial biotechnology. To illustrate AI’s real-world impact, we examine several case studies that highlight its practical applications. One of the most groundbreaking achievements is AlphaFold’s contribution to protein structure prediction. Developed by DeepMind, AlphaFold uses deep learning to predict protein structures with near-experimental accuracy, addressing a long-standing challenge in structural biology. By predicting the structures of over 350,000 proteins, AlphaFold has accelerated drug design, enabling more effective protein-ligand modeling and the development of targeted therapies. AI has also shown immense promise in combating antibiotic resistance. In a landmark study, Stokes et al. applied deep learning to screen millions of compounds, identifying Halicin—a molecule with potent activity against multi-drug-resistant bacteria. This AI-driven approach significantly reduced the time and cost traditionally associated with antibiotic development. In industrial biochemistry, AI is enhancing enzyme engineering. Ryu et al. utilized neural networks to predict EC numbers, streamlining biocatalysis and biofuel production processes. This has replaced labor-intensive trial-and-error methods, improving efficiency and scalability. AI’s impact is further evident in cancer research. Vasaikar et al. integrated multi-omics data to uncover pathways critical to cancer progression. By analyzing genomics, proteomics, and metabolomics datasets, AI identified actionable targets and biomarkers for personalized treatments, advancing precision medicine. Finally, AI is revolutionizing molecule design through GANs. Zeng et al. employed GANs to create novel molecular structures optimized for specific functions, dramatically reducing experimental trial-and-error processes and accelerating drug development timelines. These case studies collectively showcase AI’s transformative potential across multiple dimensions of biochemistry, from structural biology to drug discovery and industrial applications.

### Future research directions in AI-driven biochemistry

AI has made significant strides in the field of biochemistry, but several areas remain underexplored, presenting exciting opportunities for future research. These gaps are crucial to address in order to fully harness AI’s potential for understanding biological processes and advancing healthcare applications.

#### Integrative multi-omics modeling

Current AI tools often analyze individual omics datasets, such as genomics or proteomics, in isolation, which restricts their ability to present a comprehensive view of biological systems [41]. To truly capture the complexity of biological networks, future research should prioritize the development of AI models capable of integrating multi-omics data. These integrated models hold the potential to uncover deeper insights into disease mechanisms, identify novel therapeutic targets, and facilitate more personalized approaches to medicine.

### AI-driven protein dynamics prediction

While advancements like AlphaFold have revolutionized our ability to predict static protein structures, understanding protein dynamics is just as critical for elucidating enzyme mechanisms and drug interactions [2]. Future AI models should integrate molecular dynamics simulations to predict conformational changes and protein flexibility in real-time biological contexts. Such advancements would deepen our understanding of protein behavior under varying physiological conditions and significantly enhance drug design efforts.

### Ethical AI frameworks in biochemistry

The rapid adoption of AI in biochemistry raises important ethical concerns, particularly regarding data privacy, algorithmic biases, and the reproducibility of AI-driven findings [42]. Future research should prioritize establishing comprehensive ethical frameworks to ensure transparency and interpretability in AI models. These efforts should include developing robust guidelines for responsible data sharing, rigorous algorithm validation, and ensuring that AI applications in healthcare consistently adhere to ethical standards.

### Improved enzyme design and engineering

While AI has demonstrated its value in predicting enzyme structures, AI-predicted enzymes often require further refinement to function effectively in real-world applications [43]. Future research could focus on integrating AI-driven predictive modeling with experimental approaches, such as directed evolution, to optimize enzyme properties. Furthermore, enhancing AI’s capability to predict enzyme-substrate interactions within complex industrial processes could greatly improve the efficiency of biotechnology applications.

### AI in rare disease research

Rare diseases often lack sufficient biological data, posing a challenge for traditional research methods [44]. AI provides a promising solution by enabling the analysis of sparse datasets, identifying biomarkers, and predicting therapeutic interventions for these diseases. To advance the field, future research should prioritize the development of generative AI models capable of simulating missing data. Such models could significantly enhance research efforts, offering deeper insights into the mechanisms of rare diseases and informing potential treatment strategies.

### AI-augmented drug development pipelines

Despite the ability of AI to discover novel drug candidates, translating these discoveries into clinically approved therapies remains a significant bottleneck in drug development [8]. Future research should focus on developing AI systems that seamlessly integrate chemical synthesis, preclinical testing predictions, and clinical trial simulations. Such AI-driven pipelines could effectively bridge the gap between virtual drug discovery and experimental validation, ultimately accelerating the drug development process.

### Expanding AI in synthetic biology

Designing and optimizing synthetic biological systems remains a labor-intensive and time-consuming process [45]. AI could play a pivotal role in advancing synthetic biology by predicting the outcomes of genetic modifications and identifying optimal metabolic pathways for applications, such as biofuel production and biopharmaceutical synthesis. To maximize this potential, future research should prioritize the development of AI models that enhance these workflows, making synthetic biology more efficient, scalable, and accessible.

#### Training dataset quality and diversity

The effectiveness of AI models depends heavily on the quality and diversity of the training datasets they are built upon [46]. To improve the performance and generalizability of AI systems in biochemistry, future research should prioritize developing comprehensive, high-quality datasets that encompass a broad spectrum of biochemical phenomena. Additionally, creating AI techniques capable of learning from smaller, noisier datasets will be critical for ensuring robustness across diverse research settings. Future research directions highlight the transformative potential of AI in advancing biochemistry. Addressing current gaps will foster interdisciplinary collaboration between AI researchers, experimental biologists, and clinicians. Such partnerships will be vital for driving impactful and sustainable advancements in AI-driven biochemistry, ultimately leading to groundbreaking innovations in healthcare and biotechnology. The intersection of AI and biochemistry presents significant opportunities for future progress, with several key research areas offering exciting potential. One critical area is improving model interpretability; developing AI algorithms that produce transparent and understandable outputs will help bridge the gap between computational predictions and biochemical validation. Integrating AI with high-throughput experimental methods will also refine predictions and accelerate discovery processes, enabling seamless transitions from in silico models to *in vitro* and *in vivo* applications. Expanding AI’s role in multi-omics analysis—such as integrating transcriptomics, proteomics, and metabolomics data—can provide holistic insights into complex biological systems, unlocking breakthroughs in systems biology and personalized medicine. Moreover, applying AI in synthetic biology holds great promise; by designing and optimizing engineered metabolic pathways and novel biomolecules, AI could revolutionize industrial biotechnology and therapeutic development. Finally, addressing ethical and regulatory concerns—such as data privacy and algorithmic biases—is crucial to ensure the responsible use of AI in biochemistry. By exploring these research avenues, scientists can harness AI’s full potential to drive impactful and sustainable innovations, advancing biochemical research and its applications.

### Technical challenges and limitations

To enhance the reliability and accessibility of AI applications in biochemistry, several technical improvements are critical to advancing the field. One of the key challenges is the lack of transparency in many deep learning models, which often function as “black boxes.” This lack of clarity limits trust and usability, particularly in high-stakes applications like drug design and clinical decision-making. To address this issue, incorporating explainable AI (XAI) techniques, such as feature importance analysis and model visualization, can provide interpretable outputs. These advancements would allow researchers to validate predictions and gain deeper insights into the biochemical mechanisms at play. Another major challenge is the reliance on limited or low-quality datasets, which can introduce biases and inaccuracies into AI models. To overcome this, standardized protocols for data collection and preprocessing are essential to ensure high-quality, unbiased datasets. Furthermore, implementing data augmentation strategies—such as synthetic data generation and transfer learning—can help diversify datasets and improve model generalization. These approaches would make AI models more robust and applicable across various research domains.

As biochemistry research increasingly relies on multi-omics data—spanning genomics, proteomics, and metabolomics—AI models must evolve to handle diverse data types simultaneously. Developing multimodal AI frameworks capable of integrating and analyzing these heterogeneous datasets is crucial. Advances in transformer models and graph neural networks present promising strategies for addressing these complex, multifaceted challenges. Real-time AI applications pose another significant challenge, as many models demand substantial computational resources. To mitigate this, optimizing AI algorithms for computational efficiency is essential. Techniques, such as model compression, pruning, and federated learning could enable AI to function effectively in real-time biochemical research or clinical settings, even on compact devices like laboratory instruments or portable diagnostic tools. Integrating experimental feedback loops into AI systems could further enhance their utility. Currently, many AI models operate in isolation, with limited interaction with experimental workflows. Designing systems that incorporate real-time feedback—such as data from directed evolution in enzyme engineering—could iteratively refine predictions, improving their reliability and practical applicability in research environments. Generative AI models, such as GANs and variational autoencoders (VAEs), show great potential for molecule design but occasionally produce non-viable or irrelevant outputs. Enhancing these models by embedding biochemical constraints or leveraging reinforcement learning strategies could ensure that generated outputs are not only novel but also practical and feasible for real-world applications.

Accessibility remains a significant challenge for researchers in smaller laboratories or resource-constrained settings. High-performance AI tools often require specialized hardware and technical expertise, creating barriers to entry. To democratize AI access, there is a growing need for user-friendly, cloud-based platforms. Such platforms would enable researchers to upload their data and receive predictions without relying on extensive computational infrastructure. Open-source solutions combined with affordable subscription models could make AI more accessible to a broader audience across academia and industry.In addition, pairing AI models with experimental automation systems has the potential to streamline research workflows dramatically. Integrating AI predictions with automated platforms—such as robotic systems for high-throughput screening or automated biochemical assays—would connect AI directly to real-world validation. This integration could accelerate discovery pipelines and drive faster advancements in biochemistry. The future of AI in biochemistry is exceptionally promising, particularly with progress in areas like explainable AI, multimodal data integration, generative modeling, and computational efficiency. By overcoming these technical hurdles and fostering accessible, user-friendly platforms, AI can evolve into a more reliable and widely adopted tool for academic and industrial research. Ultimately, these advancements have the potential to revolutionize biochemistry and its applications.

## Conclusion

AI has significantly expanded the scope of biochemical research by improving predictive accuracy, automating complex processes, and reducing the time and cost associated with experimental studies. The success of AI in areas, such as drug discovery, protein structure prediction, and enzyme engineering highlights its transformative potential. However, challenges persist, particularly in the interpretability of AI models, the quality of datasets, and the ethical considerations tied to AI-driven research. Despite these hurdles, ongoing advancements in AI technologies promise deeper integration into biochemical research, driving innovations in personalized medicine, synthetic biology, and beyond. The reviewed studies indicate that AI has evolved from being a supplementary tool to becoming a core component of biochemical research. It equips researchers and industry professionals with powerful solutions to tackle complex biochemical problems. Moving forward, future research should prioritize improving the transparency of AI models and fostering their integration with experimental methodologies to further broaden the possibilities in biochemical research. Collaboration between AI experts and biochemists will be crucial in unlocking the full potential of AI. As these models become more transparent and dependable, their applications in biochemistry are expected to grow, pushing the boundaries of scientific discovery.

### Summary and outlook

Artificial intelligence has emerged as a transformative tool in biochemistry, driving innovations in drug discovery, protein structure prediction, enzyme engineering, and metabolic pathway analysis. Its ability to process and analyze vast datasets, predict complex molecular interactions, and automate labor-intensive processes underscores its value in advancing biochemical research. As the field continues to evolve, several prospects and key issues warrant attention:

1. Enhanced algorithm development: The creation of interpretable, robust, and biologically relevant AI models is critical to ensure the accuracy and reliability of predictions. This requires advances in algorithm design, incorporating domain-specific knowledge and addressing the “black box” nature of current models.
2. Data quality and accessibility: The efficacy of AI-driven research is highly dependent on the availability of diverse, high-quality datasets. Researchers should prioritize data curation, standardization, and the development of shared repositories to enhance reproducibility and accessibility.
3. Integration with experimental biochemistry: AI must work in tandem with experimental approaches to validate and refine its predictions. This interdisciplinary collaboration will ensure that AI applications remain grounded in biological realities.
4. Ethical and regulatory considerations: As AI applications in biochemistry expand, addressing ethical concerns—such as data privacy, algorithmic bias, and the societal implications of AI-driven discoveries—will be vital. Establishing clear guidelines and frameworks will support responsible innovation.
5. Applications in emerging fields: AI’s potential in frontier areas like personalized medicine, synthetic biology, and multi-omics integration presents exciting opportunities for future research. These applications promise to redefine the boundaries of biochemistry, fostering breakthroughs in both fundamental and applied sciences.

In conclusion, while AI has already significantly impacted biochemistry, its true potential remains untapped. By tackling the highlighted challenges and fostering interdisciplinary collaborations, researchers can pave the way for groundbreaking innovations, redefining the future of biochemistry in extraordinary ways.
